# Gait Analysis of Kinematic Changes After Vulpius Gastrocnemius Recession in Children With Spastic Hemiplegic Cerebral Palsy and Equinus Deformity

**DOI:** 10.7759/cureus.107640

**Published:** 2026-04-24

**Authors:** Ana Luisa Galicia-Zamalloa, Jorge Gómez-Chavarria, Rafael González-Ramírez, Víctor Hugo Sánchez-Gómez

**Affiliations:** 1 Articular Reconstruction and Arthroscopic Surgery, National Institute of Rehabilitation, Mexico City, MEX; 2 Pediatric Orthopedics, Shriners Hospitals for Children, Mexico City, MEX; 3 Pediatric Orthopedics, Hospital MAC Cuemanco, Mexico City, MEX; 4 Orthopedic Surgery, Mexican Social Security Institute, Puebla, MEX

**Keywords:** achilles tendon lengthening, cerebral palsy, equinus deformity, gait analysis, gastrocnemius recession

## Abstract

Background: Equinus deformity is a common gait abnormality in children with spastic hemiplegic cerebral palsy, resulting in altered ankle kinematics and impaired gait mechanics. Surgical lengthening of the gastrocnemius-Achilles complex aims to restore ankle dorsiflexion and improve functional ambulation.

Methods: A retrospective observational study was conducted, including pediatric patients with spastic hemiplegia and equinus deformity treated with Vulpius gastrocnemius recession. Three-dimensional gait analysis was performed preoperatively and six months postoperatively. Kinematic parameters were compared using the Wilcoxon signed-rank test.

Results: Twenty-four patients were included. Significant improvements were observed in ankle dorsiflexion at initial contact and during the swing phase, allowing restoration of heel strike and improved foot clearance. Weight-bearing distribution increased from 30% to 49%. Knee kinematics showed partial improvement. Recurrence of equinus deformity occurred in four patients.

Conclusions: Vulpius gastrocnemius recession improves ankle kinematics and gait mechanics in children with spastic hemiplegic cerebral palsy, supporting its role as an effective surgical option for equinus deformity.

## Introduction

Equinus deformity represents the most common gait abnormality in children with spastic cerebral palsy and results from spasticity and contracture of the gastrocnemius-soleus complex [1-3]. Surgical lengthening of the gastrocnemius-soleus complex is commonly performed to improve ankle kinematics and restore a plantigrade gait pattern [4,5]. However, quantitative changes in gait parameters following surgical correction remain incompletely characterized.

Three-dimensional gait analysis has become the gold standard for evaluating gait abnormalities in children with cerebral palsy and provides objective measurements of lower-limb kinematics [4]. Through quantitative assessment of joint motion throughout the gait cycle, gait analysis helps guide surgical decision-making and evaluate postoperative outcomes.

Surgical lengthening of the gastrocnemius-soleus complex is commonly performed when conservative treatment fails to correct equinus deformity. Selective gastrocnemius recession techniques, such as the Vulpius procedure, aim to increase ankle dorsiflexion while minimizing the risk of over-lengthening and loss of plantarflexion power [5].

Previous studies have demonstrated that gastrocnemius recession improves ankle kinematics and overall gait function in children with cerebral palsy [6-9]. However, the magnitude of kinematic changes following selective gastrocnemius recession remains an area of ongoing investigation.

The purpose of this study was to compare preoperative and postoperative kinematic gait parameters in children with spastic hemiplegic cerebral palsy treated with Vulpius gastrocnemius recession using three-dimensional gait analysis.

## Materials and methods

Study design

A retrospective, observational, analytical study was conducted in accordance with the Strengthening the Reporting of Observational Studies in Epidemiology (STROBE) guidelines.

Study population

Patients evaluated at the Shriners Children’s Motion Analysis Centre with a diagnosis of spastic hemiplegic cerebral palsy and equinus deformity were retrospectively reviewed. Eligible patients were identified from institutional clinical and gait laboratory databases over 10 years.

Inclusion and exclusion criteria

Postoperatively, all patients were immobilized in a below-knee cast for four weeks to maintain the correction and allow adequate soft-tissue healing. After cast removal, patients underwent a standardized rehabilitation protocol supervised by a physiotherapist.

The rehabilitation program focused on progressive restoration of ankle range of motion, strengthening of the dorsiflexor and plantarflexor muscle groups, and gait re-education. Therapy included active and passive stretching exercises, resistance training, and functional gait training. Patients attended physiotherapy sessions two to three times per week, complemented by a home-based exercise program.

Surgical technique

All patients underwent gastrocnemius recession using the Vulpius technique. This procedure consists of a transverse release of the gastrocnemius aponeurosis proximal to the musculotendinous junction, allowing controlled lengthening of the gastrocnemius-soleus complex while preserving the structural integrity of the Achilles tendon. The procedure aims to improve ankle dorsiflexion while minimizing the risk of over-lengthening and weakening of plantarflexion strength.

Postoperative management

Postoperatively, all patients were immobilized in a suropodalic cast for four weeks to maintain correction and facilitate soft tissue healing. Following cast removal, patients participated in a structured rehabilitation program that included physiotherapy focused on range of motion, muscle strengthening, and gait training.

Gait analysis

Three-dimensional gait analysis was performed at the Shriners Children’s Motion Analysis Centre using a standardized protocol. Kinematic parameters of the ankle and knee joints were recorded throughout the gait cycle. Evaluations were conducted preoperatively and at six months postoperatively under consistent laboratory conditions.

Gait patterns were classified using the Winters, Gage, and Hicks (WGH) classification system, which categorizes gait abnormalities in patients with spastic hemiplegia based on kinematic deviations [3].

Statistical analysis

Continuous variables were expressed as mean ± standard deviation. Preoperative and postoperative kinematic parameters were compared using the Wilcoxon signed-rank test due to the non-parametric distribution of the data. Statistical significance was defined as a p-value < 0.05. All statistical analyses were performed using R statistical software (R Foundation for Statistical Computing, Vienna, Austria).

Ethical considerations

The study protocol was reviewed and approved by the Institutional Review Board of Shriners Children’s Hospital (approval number CEI-2024-03). The study was conducted in accordance with the principles of the Declaration of Helsinki. Due to the retrospective nature of the study, the requirement for informed consent was waived.

## Results

Patient demographics

A total of 24 patients met the inclusion criteria and were included in the final analysis. The cohort consisted of 14 males and 10 females, with a mean age at the time of surgery of 9 years (range, 4.2-16.6 years). The mean duration of follow-up was six years.

Regarding functional status, the distribution according to the Gross Motor Function Classification System (GMFCS) was as follows: level I in 5 patients (20.8%), level II in 13 patients (54.2%), and level III in 6 patients (25%). The affected side was the left in 13 patients (54.2%) and the right in 11 patients (45.8%).

Gait pattern classification

According to the WGH classification, the most prevalent gait pattern was type III, observed in 15 patients (62.5%), followed by type IV in 6 patients (25%), and type II in 3 patients (12.5%).

Ankle kinematics

Significant improvements were observed in multiple ankle kinematic parameters following surgery (Table 1). At initial contact, ankle dorsiflexion improved from -8.75° preoperatively to -3.5° postoperatively (p = 0.014), indicating a shift toward a more physiological heel strike pattern.

**Table 1:** Changes in ankle kinematics before and after Vulpius gastrocnemius recession Statistical analysis: Wilcoxon signed-rank test. *Statistically significant (p < 0.05).

Parameter	Preoperative (mean ± SD)	Postoperative (mean ± SD)	p-value
Initial contact angle (°)	-8.75 ± 5.9	-3.5 ± 4.8	0.014*
Mean dorsiflexion during stance (°)	-10.04 ± 9.6	1.6 ± 5.9	0.059
Push-off initiation angle (°)	-4.9 ± 3.2	6.14 ± 2.4	0.01*
Toe-off angle (°)	-22.03 ± 7.5	-7.2 ± 7	0.04*
Mean dorsiflexion during swing (°)	-19.32 ± 11.4	-2.3 ± 7.5	0.002*

Similarly, the angle at the initiation of push-off increased significantly from -4.9° to 6.14° (p = 0.01), reflecting improved ankle positioning during terminal stance. The toe-off angle also showed significant improvement, from -22.03° preoperatively to -7.2° postoperatively (p = 0.04).

During the swing phase, mean ankle dorsiflexion improved markedly from -19.32° to -2.3° (p = 0.002), indicating enhanced foot clearance. Although mean dorsiflexion during stance improved from -10.04° to 1.6°, this change did not reach statistical significance (p = 0.059).

Overall, these findings demonstrate a consistent shift toward normalization of ankle kinematics across the gait cycle.

Knee kinematics

Changes in knee kinematics were more modest (Table 2). The knee angle at initial contact decreased from 33° to 30°, although this difference was not statistically significant (p = 0.16). Similarly, no significant change was observed in the knee angle at push-off initiation (8.7° vs. 14.3°, p = 0.36).

**Table 2:** Changes in knee kinematics before and after Vulpius gastrocnemius recession Statistical analysis: Wilcoxon signed-rank test. *Statistically significant (p < 0.05).

Parameter	Preoperative (mean ± SD)	Postoperative (mean ± SD)	p-value
Initial contact angle (°)	33 ± 11.4	30 ± 7.8	0.16
Push-off initiation angle (°)	8.7 ± 7.8	14.3 ± 7.5	0.36
Maximum extension during swing (°)	35.66 ± 9.0	31.2 ± 5.2	0.012*

However, a statistically significant improvement was observed in maximum knee extension during the swing phase, which decreased from 35.66° preoperatively to 31.2° postoperatively (p = 0.012), suggesting a trend toward improved limb positioning during terminal swing.

Gait cycle analysis

**Figure 1:**
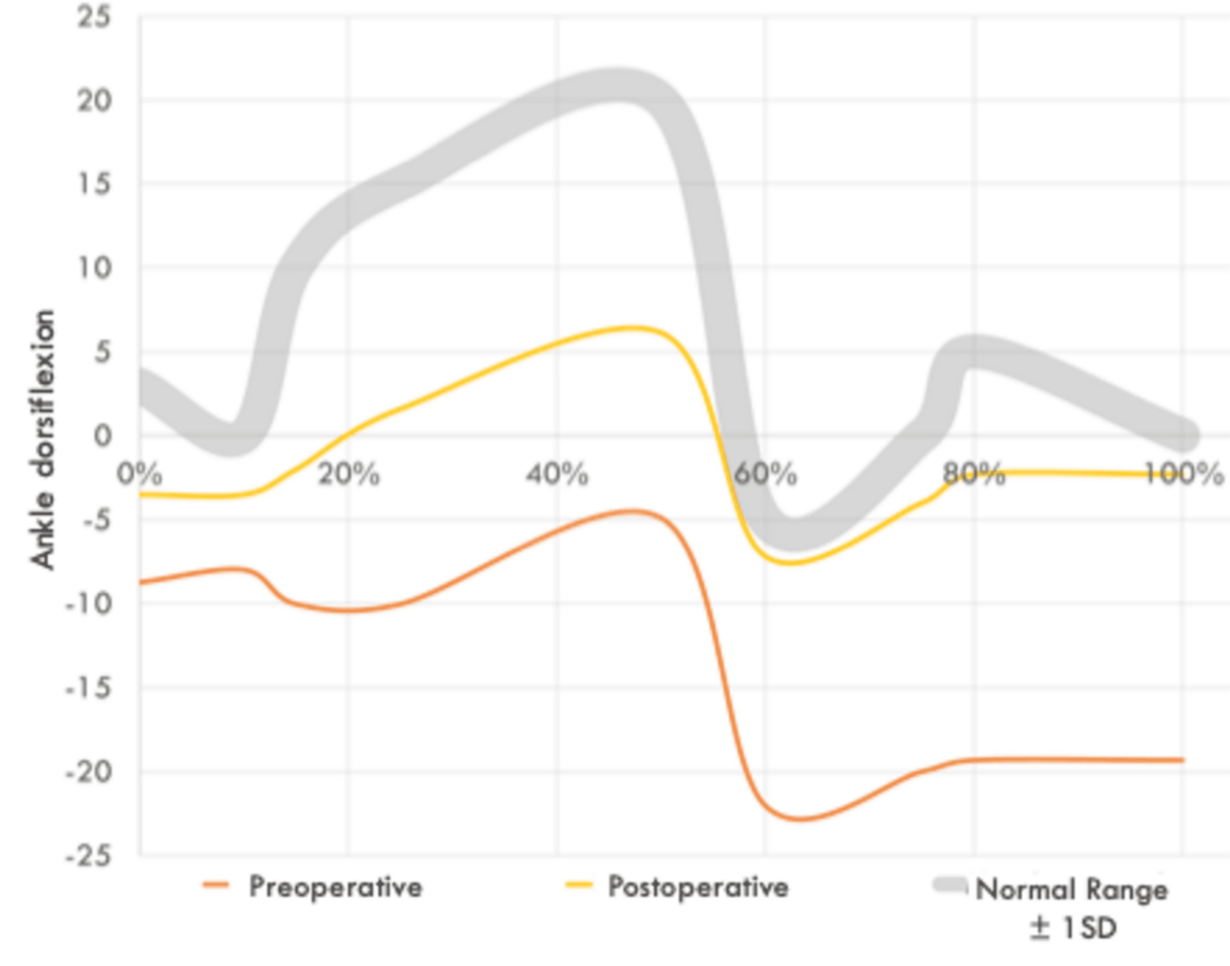
Ankle dorsiflexion during the gait cycle before and after Vulpius gastrocnemius recession in spastic hemiplegic cerebral palsy

Three-dimensional gait analysis demonstrated a shift toward improved ankle dorsiflexion throughout the gait cycle (Figure 1). Preoperatively, patients exhibited persistent plantarflexion during both stance and swing phases. Postoperatively, dorsiflexion increased, particularly during the initial contact and swing phase, approaching physiological ranges.

Representative case

**Figure 2:**
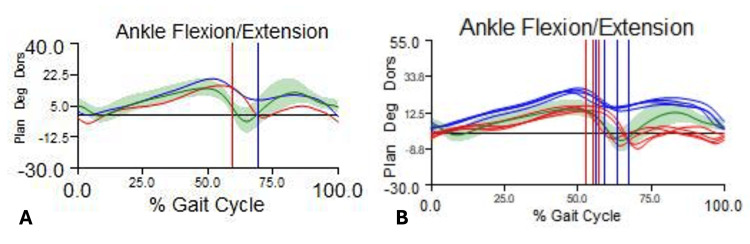
Representative case of gait kinematic changes before and after surgery (A) Preoperative analysis demonstrating increased plantarflexion throughout the gait cycle, absence of heel strike, and loss of the first rocker during initial contact. (B) Postoperative analysis at six months showing improved ankle dorsiflexion, restoration of heel strike, and re-establishment of the first rocker, with improved swing-phase clearance.

A representative case is presented to illustrate the kinematic changes following Vulpius gastrocnemius recession (Figure 2).

The patient was a child with right spastic hemiplegic cerebral palsy and equinus deformity, who underwent isolated gastrocnemius recession. Preoperative gait analysis demonstrated a persistent equinus pattern, characterized by increased plantarflexion throughout the gait cycle, absence of heel strike at initial contact, and loss of the first rocker mechanism.

Postoperative gait analysis at six months showed a clear improvement in ankle kinematics, with increased dorsiflexion at initial contact, restoration of heel strike, and re-establishment of the first rocker. Additionally, improved foot clearance during the swing phase was observed. The ankle dorsiflexion curve demonstrated a shift toward physiological patterns across the gait cycle.

Complications

Recurrence of equinus deformity was observed in four patients (16.7%) during long-term follow-up.

## Discussion

The present study evaluated kinematic gait changes following Vulpius gastrocnemius recession in children with spastic hemiplegic cerebral palsy presenting with dynamic equinus deformity. Our findings demonstrate significant improvements in ankle dorsiflexion during multiple phases of the gait cycle, with secondary improvements observed in knee kinematics. These results support the role of gastrocnemius recession as an effective surgical intervention to restore ankle biomechanics and improve overall gait function. To contextualize our findings, previously published studies evaluating gastrocnemius recession or Achilles tendon lengthening in cerebral palsy were reviewed (Table 3).

**Table 3:** Comparative outcomes of gastrocnemius recession and Achilles tendon lengthening procedures reported in the literature

Study	Patients	Procedure	Key Findings
Park et al. [5]	29 children with cerebral palsy	Gastrocnemius recession	Improved ankle dorsiflexion and restoration of heel contact
Fujita et al. [6]	38 patients	Gastrocnemius recession	Improved swing-phase dorsiflexion and gait pattern
Feng et al. [7]	42 children	Achilles tendon lengthening	Improved ankle and secondary knee kinematics
Kay et al. [10]	35 patients	Gastrocnemius recession	Improved gait with low overcorrection risk
Present study	24 patients	Vulpius technique	Improved dorsiflexion, heel strike, and swing clearance

Equinus deformity represents the most common gait abnormality in ambulatory children with cerebral palsy and is primarily caused by spasticity and contracture of the gastrocnemius-soleus complex [1,2]. Persistent plantarflexion during stance leads to forefoot initial contact, impaired shock absorption, and abnormal loading patterns throughout the lower limb. Equinus deformity is frequently associated with altered knee kinematics and compensatory gait strategies that increase energy expenditure during walking [1,2]. Consequently, correction of equinus deformity is a central component of surgical management in ambulatory patients with cerebral palsy.

Our results showed a significant improvement in ankle dorsiflexion at initial contact, which improved from -8.75° preoperatively to -3.5° postoperatively. Additionally, dorsiflexion during the swing phase improved significantly, increasing from -19.32° to -2.3°. These changes indicate restoration of heel contact and improved foot clearance during the swing phase, both of which are critical determinants of efficient gait. Restoration of heel contact during initial contact represents a critical biomechanical objective in the treatment of equinus gait, as it allows for improved shock absorption and a more stable stance phase. Similar improvements in ankle dorsiflexion following gastrocnemius recession have been reported in previous studies. Park et al. demonstrated that selective gastrocnemius recession significantly improves ankle dorsiflexion and overall gait mechanics in children with cerebral palsy while preserving adequate plantarflexion power [5]. Likewise, Kay et al. reported that selective gastrocnemius lengthening effectively restores plantigrade foot positioning during gait and improves functional mobility in ambulatory children with cerebral palsy [10].

In addition to improvements at initial contact, our study demonstrated significant increases in dorsiflexion during the swing phase of gait. Adequate ankle dorsiflexion during swing is essential for sufficient toe clearance and prevention of compensatory mechanisms such as hip hiking or circumduction. Previous gait analysis studies have shown that correction of equinus deformity significantly improves swing-phase foot clearance and reduces abnormal compensatory movements during gait [7]. Fujita et al. reported similar findings, demonstrating improved ankle kinematics throughout the gait cycle following gastrocnemius recession in children with cerebral palsy [6].

An additional finding of the present study was the improvement in knee kinematics following surgical correction of equinus deformity. Although the surgical procedure directly targets the gastrocnemius muscle, the gastrocnemius functions as a biarticular muscle crossing both the knee and ankle joints. Excessive plantarflexion can therefore influence knee mechanics through abnormal coupling between ankle and knee motion. Correction of equinus deformity may consequently reduce abnormal knee flexion patterns and improve overall lower-limb alignment during gait. Similar secondary improvements in knee kinematics following correction of equinus deformity have been reported in previous gait analysis studies [8].

The biomechanical explanation for these findings lies in the role of the gastrocnemius in controlling ankle plantarflexion while also contributing to knee flexion moments during gait. Persistent equinus can lead to abnormal knee extension during stance or altered knee flexion patterns during swing. Restoration of ankle dorsiflexion, therefore, helps normalize the kinetic chain of the lower limb, improving the coordination of ankle and knee motion during walking.

Despite the favorable kinematic outcomes observed in this study, recurrence of equinus deformity occurred in four patients during long-term follow-up. Recurrence following gastrocnemius recession has been described in previous studies and may be related to progressive muscle spasticity, growth-related changes in muscle-tendon length, or incomplete correction of the underlying deformity [8]. Long-term follow-up studies have shown that recurrence rates may increase as children grow, particularly in patients with more severe spasticity or higher GMFCS levels [11,12].

Several limitations should be considered when interpreting the findings of this study. First, the retrospective design introduces potential selection bias and limits the ability to establish causal relationships. Second, the sample size was relatively small, although comparable to many previous gait analysis studies in pediatric cerebral palsy populations. Third, postoperative gait analysis was performed at six months, which may not fully capture long-term adaptations in gait biomechanics following surgical intervention. Future prospective studies with larger cohorts and longer follow-up periods would help further clarify the long-term biomechanical outcomes of gastrocnemius recession in children with cerebral palsy.

Nevertheless, the present study provides objective quantitative evidence supporting the effectiveness of the Vulpius gastrocnemius recession in improving ankle dorsiflexion and overall gait kinematics in children with spastic hemiplegic cerebral palsy. The use of three-dimensional gait analysis allowed for a detailed assessment of joint motion throughout the gait cycle and provides important insight into the biomechanical effects of selective gastrocnemius lengthening procedures.

## Conclusions

Vulpius gastrocnemius recession is an effective surgical procedure for the correction of dynamic equinus deformity in children with spastic hemiplegic cerebral palsy. The results of this study demonstrate significant improvements in ankle dorsiflexion during multiple phases of the gait cycle, including initial contact and swing phase, leading to restoration of heel contact and improved foot clearance during walking.

In addition to the improvements observed in ankle kinematics, secondary changes in knee motion were identified, supporting the biomechanical interdependence between ankle and knee joints in the lower-limb kinetic chain. These findings highlight the importance of selective gastrocnemius lengthening in optimizing gait mechanics while minimizing the risk of overcorrection. Three-dimensional gait analysis proved to be a valuable tool for objectively evaluating surgical outcomes and quantifying biomechanical changes following correction of equinus deformity. Overall, the Vulpius gastrocnemius recession represents a reliable surgical option for improving gait biomechanics and functional ambulation in children with spastic hemiplegic cerebral palsy.
